# EYE‐CODE Protocol for the Nonophthalmologist for Treatment of Retinal Artery Occlusion After Intra‐Arterial Injection of Soft‐Tissue Fillers: 2025 Update

**DOI:** 10.1111/jocd.70336

**Published:** 2025-07-09

**Authors:** Sheila Barbarino, Saami Khalifian, John Fezza

**Affiliations:** ^1^ Barbarino Surgical Arts Austin Texas USA; ^2^ SOM Aesthetics Encinitas California USA; ^3^ Center for Sight Sarasota Florida USA

**Keywords:** intraocular, ischemic, protocol, retinal artery occusion, soft‐tissue filler, vision loss

## Abstract

**Background:**

Vision loss following a facial esthetic procedure with soft‐tissue fillers is an event for which there is only a short treatment window to restore blood flow before irreversible ischemic damage occurs.

**Objective:**

To provide a current standardized treatment approach for the management of retinal artery occlusion after intra‐arterial injection of soft‐tissue fillers.

**Methods:**

This document provides an updated, simple, and evidence‐based protocol for acute treatment in the office setting using the acronym EYE‐CODE: EYE (I call retinal referral center), C (check vision and optic nerve function), O (ocular massage), D (decrease intraocular pressure), and E (erase filler).

**Results:**

The EYE‐CODE protocol assists esthetic physicians to determine the extent of vision loss and provides an initial treatment strategy using medications likely to be available in most practices. This includes agents that reduce intraocular pressure, improve retinal perfusion, and potentially dissolve the filler embolus and any associated blood clots. Suggestions are also made for treatments that can be continued by an emergency ophthalmologist.

**Conclusion:**

The EYE‐CODE 2025 update provides physicians with a comprehensive reference for the management of retinal artery occlusion caused by soft‐tissue filler injections.

## 1 Introduction

Retinal artery occlusion after a soft‐tissue filler procedure is a rare event. The incidence has, however, increased in the past 5 years, most likely as a result of the everincreasing popularity of filler injections and the varied skill level of injectors. In a 2024 review of the worldwide literature, case reports had risen from a total of 146 to 511 cases over the 5‐year period from 2018 to 2023 [1]. Vision loss has also been reported after facial injections of platelet‐rich plasma [2]. Needle versus cannula is an ongoing debate in terms of injection safety. A survey of 370 US dermatologists, which collected data on rates of vascular occlusion with US FDA‐approved injectable fillers over a 10‐year period, reported that while rare, occlusions were more frequent following injections with needles than microcannulas [3]. However, ocular occlusions have been reported with both instruments [1] and while 22G and 25G cannulas require greater forces for intra‐arterial penetration compared with correspondingly sized needles, 27G cannulas and 27G needles have a similar force requirement [4]. The survey results also revealed that more experienced injectors (more than 5 years in clinical practice) were 71% less likely to cause an occlusion than those with less experience [3].

As demand for filler injections increases, so too will the demand for injectors. Those new to the field will inevitably have less training and experience.

Considering the critical nature and time‐sensitive aspect of retinal artery occlusion in typically healthy patients undergoing optional treatments, it is crucial for medical professionals to be well versed in anatomically risky regions, initial indicators, and protocols for addressing filler‐induced vision loss. Contemporary research indicates that irreversible damage from ischemia could occur within a quarter of an hour [1, 5]. However, depending on the occlusion's location and magnitude, there may still be potential for salvaging some tissue and visual function even after this critical period. Consequently, the protocol for addressing retinal occlusion should be implemented for all affected individuals, regardless of the time elapsed since the incident. It is imperative that all physicians performing injections have a predetermined action plan in place to manage such emergencies.

While ophthalmologists and oculoplastic surgeons are the most qualified to deal with retinal artery occlusions should they occur, only around 1% of facial esthetic treatments are performed by these providers, compared with 33% of plastic surgeons and 32% of dermatologists [6]. For this reason, the authors published a standardized approach for the treatment of retinal artery occlusion in 2022 [7]. Since this paper was published, there have been several important developments in the field, and this 2025 update reviews the new evidence and incorporates strategies with demonstrated effectiveness into a revised EYE‐CODE protocol. As before, the paper has been authored by a team of ophthalmologists and a dermatologist with expertise in treating both filler‐induced occlusions and nonfiller‐related retinal artery occlusions.

## 2 Anatomy and Pathogenesis

**FIGURE 1:**
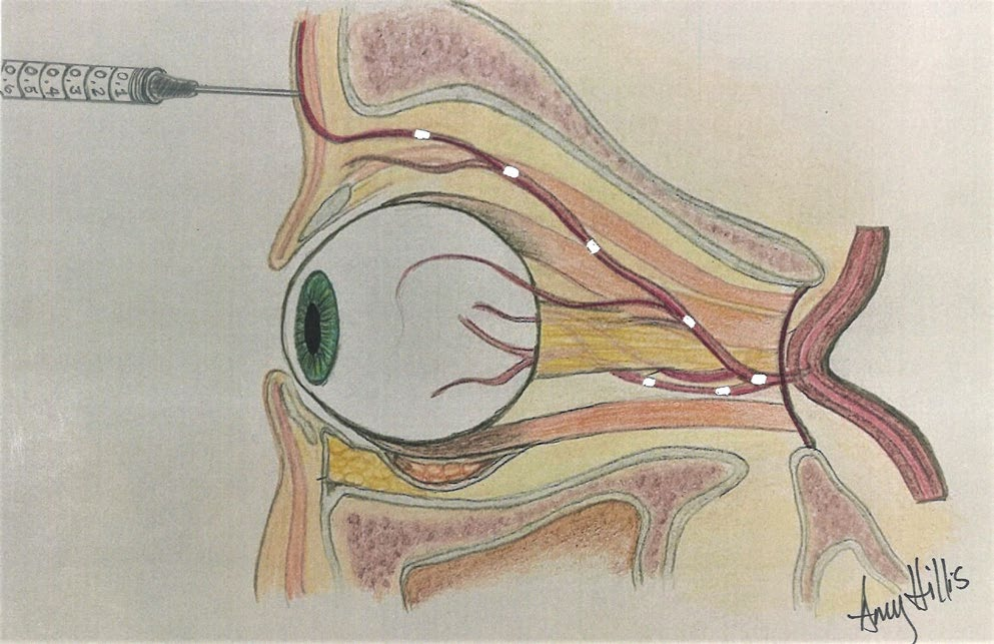
Devastating visual loss can result from inadvertent injection of filler into peri‐ocular branches of the carotid artery that anastomose with the posterior intra‐orbital circulation. Superficial injection of filler into the supratrochlear artery is shown with a direct pathway connecting back to the central retinal circulation. This can result in optic nerve ischemia and blindness.

The neural tissue of the retina relies heavily on sufficient blood flow, and occlusion of retinal arteries and resulting ischemia can lead to significant visual impairment or vision loss. Following a cosmetic procedure, a widely accepted mechanism for retinal artery occlusion—a form of a retinal stroke—is accidental intra‐arterial injection into a distal branch of the ophthalmic artery, for example, supraorbital, supratrochlear, and dorsal nasal arteries (Figure 1). These arteries form part of the internal carotid artery system and supply the glabellar, forehead, and nasal regions. Numerous anastomoses exist between the internal and external carotid systems, such as those between the dorsal nasal and angular arteries, or the supraorbital artery and superficial temporal artery. There is also significant variation in anastomoses between individuals. Temple injections, in particular, are becoming increasingly popular and have been linked to a number of cases of filler‐related vision loss [1]. The risk is increased in sites of previous trauma or surgery due to changes in the natural vascular system and a decrease in collateral circulation (e.g., in sites of previous rhinoplasty, childhood traumas, previous browlifts) [8].

If the injection pressure exceeds mean arterial pressure, filler material may enter an artery and be advanced proximally into the ophthalmic artery, opposing its natural flow direction. Releasing the pressure on the syringe restores normal antegrade blood flow, which may then result in the filler traveling distally and occluding the ophthalmic artery as well as its intraocular branches, resulting in ophthalmic artery occlusion (OAO), central retinal artery occlusion (CRAO), or branch retinal artery occlusion (BRAO).

Other research has shown that anastomoses between (and within) the facial and ophthalmic angiosomes consist of two types. True anastomoses are of constant caliber, while choke vessels have reduced caliber during normal vascular supply but have the ability to dilate and increase blood flow when there is demand [9]. Choke vessels can limit the ability of filler to spread to adjacent angiosomes through a protective vasoconstrictive reflex. However, this constriction may also contribute to decreased tissue perfusion in cases of filler‐induced vascular occlusion, potentially worsening the degree of ischemia.

As mentioned above, anastomoses may allow filler injected into an artery of the external carotid system to enter the internal carotid system and subsequently enter the ophthalmic artery [10]. Some individuals may be more susceptible to filler emboli. Recent studies have found that approximately one‐third of patients with internal carotid artery stenosis exhibit anterograde flow from the external carotid artery to the ophthalmic artery, potentially creating a pathway for small filler particles to enter the ophthalmic circulation [11]. Another potential higher risk group is those with patent foramen ovale (PFO). This common congenital heart disease is characterized by a right‐to‐left atrial shunt. Development of BRAO in a PFO patient following filler injection led investigators to speculate that following injection into the nose, the HA filler had entered the venous system and traveled through the superior vena cava to reach the right heart. As a result of the right‐to‐left atrial shunt, the filler was thought to have then directly entered the left heart and flowed into the systemic circulation through the internal carotid artery [12].

OAO results in the most severe loss of vision due to ischemia of both the inner and outer retina. CRAO also results in severe loss of vision as the central retinal artery supplies the inner retina. The presence of a cilioretinal artery may slightly improve prognosis following OAO and CRAO. This artery, which is present in 5%–30% of the population, allows the occlusion in the central retinal artery to be bypassed [13, 14]. Sight impairment after BRAO may be limited to partial vision loss. Prognosis following all types of occlusion is also influenced by soft tissue filler type, injection volumes, product cohesivity, and size of the particles [1].

## 3 Treatment Window

Occlusion of the retinal artery represents a critical ocular emergency, characterized by immediate and total loss of vision, which can become permanent due to the death of retinal cells [5, 15]. Despite the limited timeframe for intervention, efforts should always be made to dislodge the obstructing material and reestablish blood flow to the retina. It is important to note that even when a patient seeks medical attention outside the recognized intervention period, the arterial blockage may not always be absolute, or there might be intermittent periods of blood flow restoration.

## 4 Updated EYE CODE—Systematic Protocol for Acute Vision Loss

**FIGURE 2:**
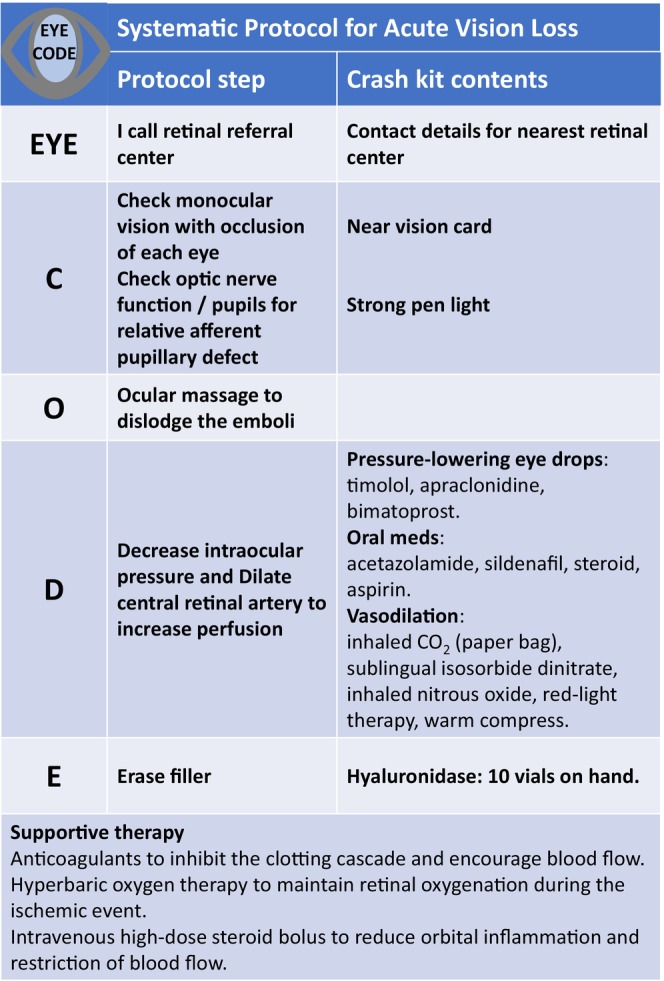
EYE‐CODE protocol.

EYE CODE (EYE = I call my retinal referral center, Check vision and optic nerve function, Ocular massage, Decrease intraocular pressure, Erase filler) (Figure 2).

### 4.1 C = Check Vision and Optic Nerve Function

Vision checks should be conducted with the patient wearing their usual vision correction aids. When examining monocular vision and performing visual field tests, the non‐tested eye should be covered. Vision is recorded using a standardized scale as follows: count fingers (CP), hand motion (HM), light perception (LP), no light perception (NLP). Snellen acuity is measured using a near vision acuity card or available alternatives like a phone app or printed material.

The relative afferent pupillary defect (RAPD) test is crucial and should be performed in a dimly lit room. The examiner alternates a light source between the affected and unaffected eyes, observing pupillary responses. Under normal conditions, both pupils constrict when light is shone into either eye. However, with NLP, the pupil of the affected eye will only constrict when light is shone into the unaffected eye. When the light is shifted to the NLP eye, both pupils dilate.

These basic visual assessments provide valuable information for ophthalmologists upon patient transfer. Studies show that initial visual acuity is a strong indicator of long‐term visual improvement [1]. For example, in cases of CRAO, around 4 out of 5 patients experience visual acuity reduced to counting fingers or worse [16].

CRAO is typically identified during a dilated eye exam, which reveals a characteristic red spot at the center of the macula surrounded by a pale retina caused by reduced blood flow. BRAO presents as a region of superficial retinal whitening that follows the path of the obstructed blood vessel.

### 4.2 O = Ocular Massage (Very Important Step)

Ocular massage should be performed with the patient lying in a supine position. The thumb is used to apply direct pressure to the affected eye through the closed eyelid using a sequence of 5 s of pressure followed by 10 s of release. The sequence is continued for 5 min and can be repeated three times with vision checks in between. Ocular massage dilates the retinal arteries and causes intraocular pressure (IOP) to fluctuate, which may rattle and displace the embolus [17].

### 4.3 D = Decrease Intraocular Pressure and Dilate Central Retinal Artery

Acute treatment is focused on dislodging the occluding material and enhancing visual results. The following approaches have all been employed with varying degrees of success to achieve this goal. The authors recommend all esthetic practices are equipped with a retinal artery occlusion “crash kit.”

#### 4.3.1 Pressure‐Lowering Eye Drops

Several eye drops commonly used to lower IOP in glaucoma patients, including acetazolamide, timolol, apraclonidine, and bimatoprost [18, 19], can be utilized to increase blood flow in cases of CRAO. Although their onset of action may occur beyond the suggested treatment window, their use is still encouraged. Administer the drops 1 min apart every 15 min until the patient is evaluated by an ophthalmologist.

#### 4.3.2 Oral Medications to Lower IOP

The glaucoma treatment acetazolamide is also available in tablet form. While it has not been evaluated in the management of CRAO, significantly lower IOP after cataract surgery has been observed following prophylactic administration compared with administration post‐surgery or no administration [20].

Nitrates act to reduce venous pressure and have a mild dilating effect on arteries. This may create a pressure gradient that propels the embolus distally in the retinal circulation. Other options to dilate the retinal arterial circulation include the oral agents sildenafil (Viagra), tadalafil (Cialis), and vardenafil (Levitra). All the above should be accompanied by the administration of 325 mg chewable aspirin to help prevent blood clotting upstream of the embolus. Anticoagulants such as clopidogrel or low‐dose heparin should also be provided to inhibit clotting and encourage blood flow to the compromised area.

#### 4.3.3 Dilating the Ophthalmic Circulation

Vasodilation may be promoted by having the patient rebreathe into a paper bag for approximately 10 min every half hour. This approach is based on the premise that carbon dioxide induces arterial dilation, which could help dislodge the clot [21]. Inhaled nitrous oxide (N_2_O) alongside other measures may improve livedo reticularis and capillary refill [22]. Warm compresses may also help enhance blood flow.

### 4.4 E = Erase Filler

For cases of CRAO caused by hyaluronic acid (HA) filler injection, approximately 3 cc of hyaluronidase is administered into the affected area and into the supraorbital or supratrochlear foramina at 15‐min intervals. In the presence of skin changes such as livedo reticular, capillary refill should be checked. Hyaluronidase should be continued for 4 cycles or until capillary refill is improved or vision returns.

Retrobulbar injection of hyaluronidase is an option if vision is not restored. The theory is that placing the hyaluronidase behind the eye as close as possible to the ophthalmic and retinal arteries may offer the best chances of rapid restoration of vision. Success rates with retrobulbar injection are variable [23, 24, 25]; however, it is also associated with inherent risks (retrobulbar hemorrhage, optic nerve damage) which the injector should consider before deciding in which cases the procedure is necessary. A further consideration is that highly concentrated hyaluronidase would be the preferred reversal agent, which may not be available in all countries, including the USA. Injectors are advised to practice this procedure on a cadaver—refer to the diagram for guidance on performing retrobulbar injections (Figure S1).

**FIGURE 3:**
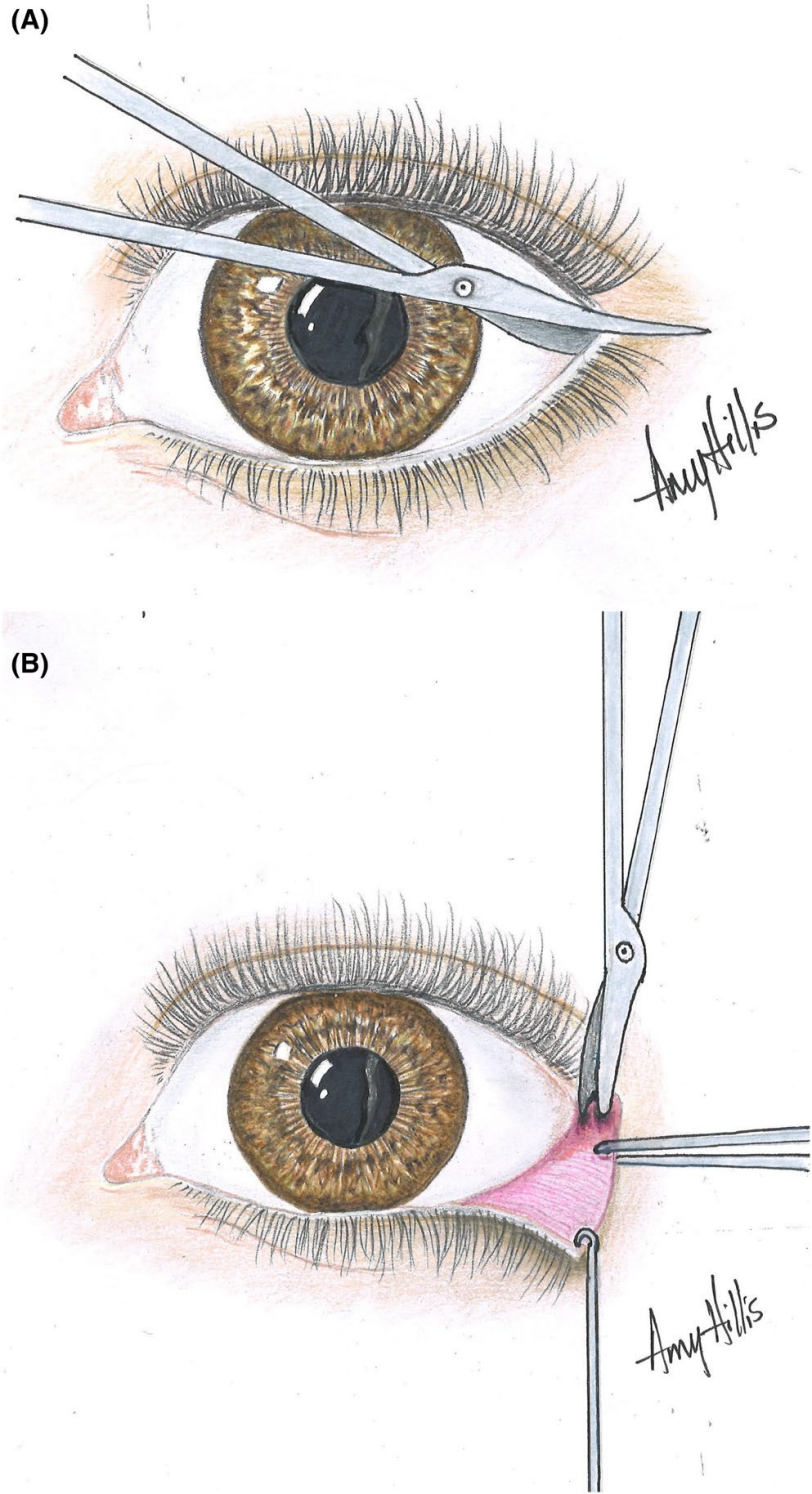
Canthotomy/cantholysis: Release of the lateral eyelids from their bony attachment allows a much greater volume of hyaluronidase to be placed into the confines of the orbit. (A) The lateral lids are anesthetized with local anesthesia and then a canthotomy is performed to separate the upper from the lower lateral lid. (B) If this maneuver does not create the desired decompression result a cantholysis must follow to release the lower canthal attachments and thus free the lid fully from the bone.

The success of retrobulbar hyaluronidase injection, as discussed by DeLorenzi, hinges on several key factors: the timing of the intervention, the embolus location, and the dosage of hyaluronidase used [25]. However, the volume of hyaluronidase is constrained by the limited capacity of the orbit, which is a confined space of about 30 cc. To address this limitation, one option is to provide an initial injection of up to 7 cc of hyaluronidase retrobulbarly, accompanied by a canthotomy and cantholysis. This procedure, carried out under local anesthesia, releases the lateral eyelids from the orbit, enabling the administration of a substantially larger volume of hyaluronidase around the ischemic area in the posterior orbit (Figure 3).

The authors recognize that many nonophthalmologists would not be comfortable performing retrobulbar injection or cantholysis. An alternative approach is percutaneous cannulation of the supraorbital or supratrochlear arteries. This method is less invasive than the retrobulbar technique and has led to immediate vision recovery in two reported cases [26]. Anatomical and ultrasound findings indicate that the supratrochlear artery follows a more superficial path and has a larger diameter than the supraorbital artery, making it potentially the better option for cannulation [27].

Other supportive treatments should include hyperbaric oxygen therapy (HBOT). This is because oxygen passively diffuses from the outer retina to the inner retina, which, if increased with HBOT, may allow retinal oxygenation to be maintained during the ischemic event and until blood flow is restored [28]. A recent case report documents complete vision recovery after the implementation of HBOT [29]. The treatment was started 10 h after the injection of HA filler into the glabellar region, which had caused immediate vision loss. Hyaluronidase and ocular massage had no effect, but the patient's vision started to improve after the first HBOT session and was completely restored after three sessions. HBOT should be considered an adjuvant treatment that allows some direct diffusion of oxygen to otherwise ischemic retinal tissues and buys time for the central retinal artery to recanalize. Administration of a high‐dose intravenous steroid bolus, such as methylprednisolone, should also be considered to help reduce orbital inflammation and improve blood flow [30].

## 5 Treatments at Retinal Referral Center

### 5.1 Thrombolytic Treatment (Tissue Plasminogen Activator [tPA])

It is now thought that when injected filler material blocks or reduces blood flow through a vessel, platelet aggregation and endothelial damage occur. Thus, even if hyaluronidase were to successfully lyse the HA thrombus, the vessel may still be occluded by platelets and fibrin. Research in animal models has shown that a combination of perivascular hyaluronidase and tPA is more effective than either agent alone for re‐perfusing an occluded vessel within the first 4 h after the event [31]. The rationale is that the tPA allows more of the hyaluronidase to reach the HA emboli.

Recent emergency protocols for CRAO are incorporating intravenous or intra‐arterial tPA treatment to treat vision loss within a similar time frame (4.5 h) [32]. All available tPA agents are serine proteases that cleave plasminogen into active plasmin; examples include alteplase, tenecteplase, and urokinase. Case reports have demonstrated notable vision restoration in patients who experienced complete blindness due to fillers, following the combined use of intra‐arterial hyaluronidase injections and tPA into the ophthalmic artery, particularly when patients are treated within the 4.5 h time frame [33, 34, 35, 36]. The 4.5 h time window is based on use of tPA in acute ischemic stroke where it has been shown to restore cerebral blood flow in some patients with improvement or resolution of neurologic deficits [37]. The optimal time window for use of these agents following retinal occlusion is not known, but as CRAO is essentially a retinal stroke [32] it seems reasonable to pursue treatment within this timeframe until further data become available. Intra‐arterial tPA administration requires smaller doses to achieve a therapeutic effect and may allow for a longer treatment window (6 h) [32]. Data from retrospective controlled trials support use of intra‐arterial tPA compared with standard therapy alone [38]. A recent pilot program installed optical coherence tomography cameras in emergency departments associated with stroke centers combined with remote consultation by retina specialists. This protocol allowed more rapid diagnosis of acute CRAO and intervention with intra‐arterial tPA, with clinically significant improvements in visual acuity [39].

### 5.2 Hyperosmotic Agents

Hyperosmotic agents create an osmotic gradient leading to a reduction in the water content of the eye and a drop in IOP. This effect can be achieved by administering intravenous mannitol 20% (1 g/kg) infused over a period of 30 to 60 min [40].

### 5.3 Intravenous Prostaglandin E1

Two retrospective studies have examined the effects of intravenous prostaglandin E1 (PGE1) infusion in patients with acute nonfiller CRAO [41, 42]. Significant improvements in vision were reported without any systemic adverse events.

### 5.4 Ischemia Reperfusion Injury

Prompt revascularization and restoration of blood flow is the cornerstone of ischemia management. Although restoring the delivery of oxygen and nutrients and clearing harmful by‐products of cellular metabolism is crucial, reperfusion may also lead to a sudden local increase in reactive oxygen species and proinflammatory neutrophils, which may worsen the ischemic injury, leading to ischemia reperfusion injury (IRI). To prevent IRI, the blood flow must be restored before the damage caused by the initial ischemic event becomes a significant contributor to overall tissue injury [43].

Strategies to avoid IRI focus on decreasing the formation of reactive oxygen species, preventing inflammatory mediators from binding to the endothelium, and decreasing neutrophil activation. If started promptly, HBOT can help reduce the harmful effects of reperfusion in affected tissues by regulating inflammation, enhancing microcirculation, preserving metabolic activity, and limiting oxidative damage. A suggested treatment protocol is 100% O_2_ at 2.8 atm for 90 min twice daily for 3 days. Steroids also have a protective role against IRI, including intravenous methylprednisolone or oral dexamethasone. Several other potential strategies may also reduce oxidative stress and IRI. Pioglitazone has been demonstrated to decrease NF‐κB activation and reduce apoptosis in a rat model of retinal ischemia/reperfusion injury [44]. In acute myocardial infarction patients, adenosine administration prior to reperfusion has proved beneficial for reducing the pathophysiological processes that lead to IRI [45]. It may therefore also have benefits for reducing retinal IRI. Hydrogen sulfide has been shown to reduce IRI in various organs and tissues, primarily by decreasing inflammation and suppressing apoptosis and oxidative stress [46]. It should be noted that optimal timing for all of these potential mechanisms for preventing IRI is prior to reperfusion, before the pathophysiological cascade leading to IRI is underway.

## 6 Medicolegal Implications

Vision loss after esthetic injections is an extremely rare occurrence relative to the large number of procedures performed, but all patients should be informed of the risk and it must be on consent forms. Nonetheless, a significant number of malpractice claims involving injectable facial fillers—both in the USA and internationally—have alleged a lack of informed consent [47, 48, 49].

In the event of a visual complication, the injecting physician must assume full responsibility for notifying the emergency facility about the patient's imminent arrival. This notification should include comprehensive details such as the time of injection, onset of symptoms, any postevent interventions undertaken, duration of transfer delay, and all pertinent medical history.

There are also important administrative procedures that need to be followed, which will vary from country to country. The product manufacturer should be notified of the adverse event, including the specific product (s) used, their volumes, and all procedural details. Additionally, the national medical device authority (such as the FDA's Medical Device Reporting system in the USA) must be informed.

A consensus document on preventing and treating filler‐induced vision loss outlines several administrative steps that injectors should adhere to [50]. This will involve notifying the appropriate medical indemnity provider and supplying accurately recorded details of all events leading up to the incident, as well as the interventions or actions taken. Additionally, all communications between the specialist emergency facility, the patient, and the injector should be thoroughly documented.

## 7 Discussion

Although exceedingly uncommon, ocular complications from cosmetic filler injections can have devastating consequences, with only a brief interval for intervention to mitigate damage and preserve vision. The majority of esthetic practitioners lack firsthand experience in managing acute vision loss, potentially leading to decision‐making challenges in high‐pressure scenarios with significant legal implications. Consequently, it is imperative for all cosmetic injectors to be thoroughly familiar with a protocol for addressing accidental intravascular filler administration. A comprehensive approach, with initial interventions administered by the aesthetic practitioner, offers the best chance for visual recovery. To support this process, the authors have developed a simple protocol (EYE CODE) for immediate management that does not require specialized training.

It is recommended that cosmetic practitioners establish a relationship with the nearest ophthalmology referral center prior to any emergencies. It is important that ophthalmologists to whom patients are referred are comfortable managing filler‐induced ocular complications. Practitioners cannot assume that this is always the case and should open discussions with the referral center prior to the need arising. This proactive measure ensures both parties are well prepared should an urgent situation arise necessitating their collaboration.

Immediate treatment in the practitioner's facility should commence with the least invasive options, beginning with ocular manipulation, followed by reducing intraocular pressure through topical and oral medications, alongside agents aimed at dilating ophthalmic and central retinal blood vessels.

New options to consider include low‐dose heparin to inhibit the clotting cascade and encourage blood flow, and intra‐arterial or intravenous injection of tPA in concert with hyaluronidase to allow more of the latter to reach the HA emboli. Intravenous tPA is the current standard treatment for acute ischemic stroke, and as CRAO is effectively a retinal form of stroke, there is strong justification for applying the same therapeutic approach. HBOT has an important adjunctive role in treating retinal ischemia within the first 12–24 h following the occlusion event, which should encourage aesthetic clinics to make contact with local HBOT centers for rapid referral should the need arise. Inhaled nitrous oxide can also be included as an adjunctive therapy if available.

Consideration should also be given to the possibility of IRI and steps taken to avoid it by implementing preventive treatments before reperfusion. Treatments that may ameliorate the damaging effects of reperfusion, that is formation of reactive oxygen species, include HBOT and intravenous steroids. Some data show pioglitazone and adenosine may also be of value.

Although there may be insufficient evidence to favor one treatment over another for acute nonfiller retinal artery occlusion [51], a stepwise protocol that integrates both medical and mechanical interventions may provide the best chance of restoring retinal circulation [52]. This structured approach has demonstrated greater success compared to nonsystematic methods in managing acute nonarteritic CRAO [52].

It is essential that injectors are aware of all the treatment options available. Most filler‐induced CRAO are recognized early as they occur almost immediately when the patient is still in the injector's practice. Systems of care should now focus on prioritizing the prompt triage of CRAO for emergency medical treatment, aiming to restore retinal perfusion within 90 min.

The authors believe that future directions may mimic current hospital stroke protocols [53]. This would involve early identification of the filler‐induced CRAO by the provider and urgent transfer to an on‐call team at the nearest hospital. This may challenge current thinking, but if enacted, may enhance the opportunity for visual recovery. The team of providers at the hospital would include the injector for the history of the event, an ophthalmologist for serial eye exams, and an interventional radiologist. The radiologist would deliver urgent and precise intra‐arterial injections of clot‐busting agents such as tPA and hyaluronic acid reversal agents such as hyaluronidase guided to the patient with sudden vision loss. An interventional radiologist would be beneficial as the drugs can be placed directly in the orbital system under radiologic guidance, and this avoids “blindly” injecting the arteries of the face as others have recently suggested. Intraocular pressure‐lowering drops and oral medicine can all be given at the hospital under the supervision of a team of experts. This would require a preplanned protocol at the hospital to have these drugs available in their formulary and the team ready and versed in the emergency protocol. The ophthalmologist would be required for serial vision and retina exams, including retinal imaging. Although this may seem cumbersome and daunting, current stroke protocols have evolved to enact similar measures. If filler‐induced blindness can be reversed or treated, this would justify placing these types of protocols in place at hospitals nationally. Our “vision” of this hospital on‐call approach may, just in fact, save vision.

## 8 Conclusion

Swift, multipronged interventions are essential to prevent or minimize vision impairment in cases of filler‐induced retinal artery blockage. Despite its rarity, all cosmetic injection practitioners should be prepared for this possibility and maintain an emergency kit of treatments. This current, clinic‐based protocol serves as a comprehensive guide for appropriate management strategies.

## Author Contributions

**Sheila Barbarino:** conceptualization, writing original draft. **Sheila Barbarino, Saami Khalifian, John Fezza:** screening and data extraction, writing – review and editing. All authors have reviewed and approved the article for submission.

## Disclosure

The authors have the following to disclose: Dr. Barbarino is a consultant and speaker for Merz, Galderma, Benev, Skinceuticals, Rohrer Aesthetics, Monarch, Promotalia, Moxi, and Sinclair Pharmaceuticals. Dr. Fezza discloses the following: Allergan (Consultant, Advisor, Speaker, FDA Trials), Revance Therapeutics (FDA Trials), Upneeq (Speaker, Advisor, FDA Trials), Ariessence (Advisor), Navaclick (Advisor), Evolus (Advisor), Nordic Pharma (Patent Holder, Speaker, Advisor). Dr. Khalifian is a consultant, speaker, and principal investigator for Allergan and Benev. He is a consultant and speaker for Merz.

## Conflicts of Interest

The authors declare no conflicts of interest.

## Supporting information


**Figure S1.** Proper retrobulbar injection technique for delivering hyaluronidase into the ischemic area behind the affected eye. (A) Sagittal view demonstrating the use of a 23‐gauge Atkinson Needle or a 25‐gauge 1½ inch needle inserted over the bony orbital rim and advanced to the intraconal (inside the extraocular muscle cone) space. This allows the hyaluronidase to be delivered closer to the area of vascular insult around the compromised optic nerve. (B) Frontal view of the retrobulbar injection showing the needle insertion point is at the lateral 2/3s of the orbit and just above the orbital rim. The needle is directed posteriorly and medially following the natural shape of the cone‐shaped orbit.

## Data Availability

Data sharing is not applicable to this article as no new data were created or analyzed in this study.
