# Supplementary figures and images for: EYE‐CODE Protocol for the Nonophthalmologist for Treatment of Retinal Artery Occlusion After Intra‐Arterial Injection of Soft‐Tissue Fillers: 2025 Update
Source: J Cosmet Dermatol. 2025 Jul 9;24(7):e70336. doi: 10.1111/jocd.70336 (PMC12239549; doi:10.1111/jocd.70336)

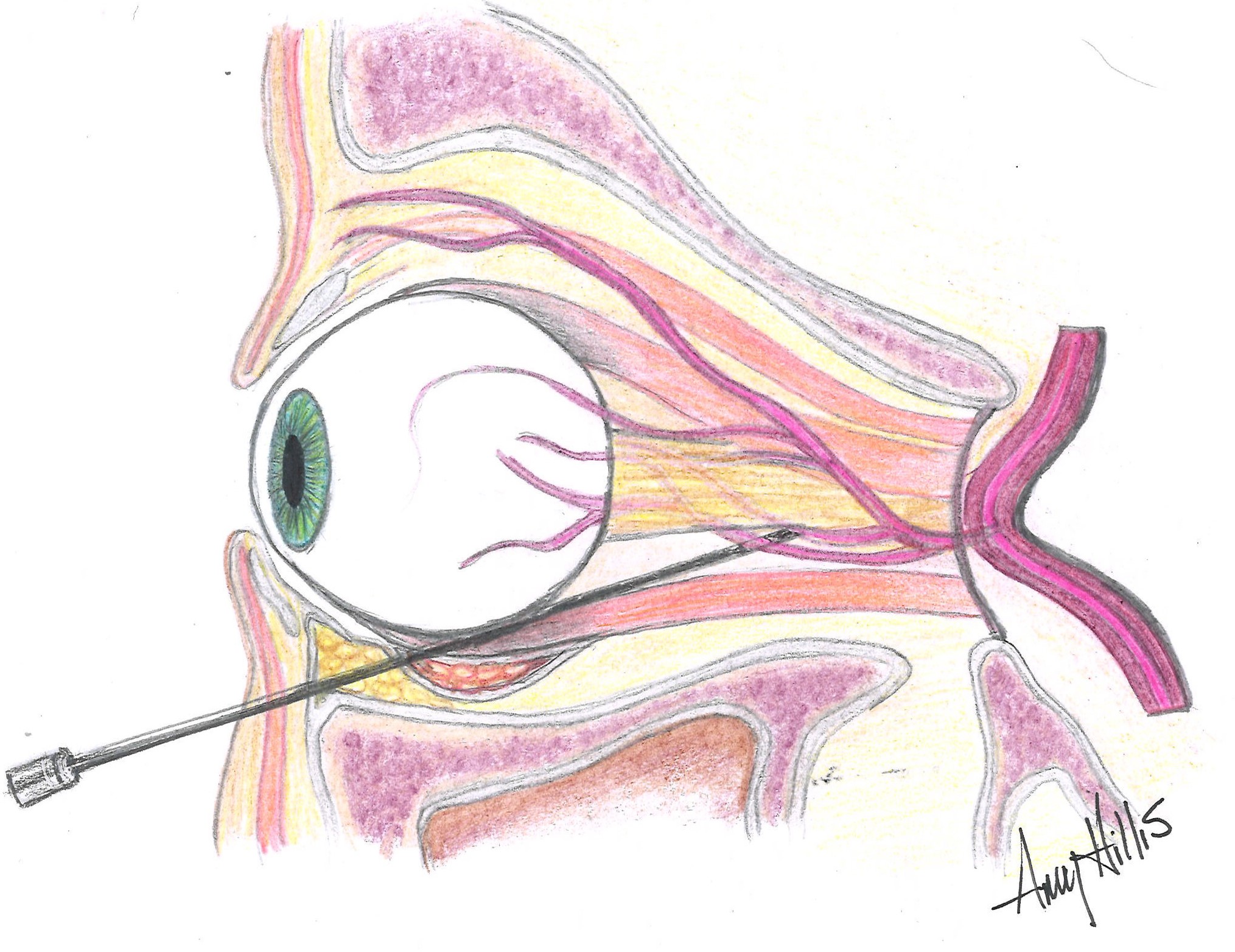

Supplement: Supplementary file 1 — Figure S1. Proper retrobulbar injection technique for delivering hyaluronidase into the ischemic area behind the affected eye. (A) Sagittal view demonstrating the use of a 23‐gauge Atkinson Needle or a 25‐gauge 1½ inch needle inserted over the bony orbital rim and advanced to the intraconal (inside the extraocular muscle cone) space. This allows the hyaluronidase to be delivered closer to the area of vascular insult around the compromised optic nerve. (B) Frontal view of the retrobulbar injection showing the needle insertion point is at the lateral 2/3s of the orbit and just above the orbital rim. The needle is directed posteriorly and medially following the natural shape of the cone‐shaped orbit. [file JOCD-24-e70336-s001.jpg]
